# Neuronal Mitochondrial Dysfunction and Bioenergetic Failure in Inflammation-Associated Depression

**DOI:** 10.3389/fnins.2021.725547

**Published:** 2021-11-01

**Authors:** Angela Maria Casaril, Robert Dantzer, Carlos Bas-Orth

**Affiliations:** ^1^Department of Medical Cell Biology, Institute for Anatomy and Cell Biology, Heidelberg University, Heidelberg, Germany; ^2^Laboratories of Neuroimmunology, Department of Symptom Research, University of Texas MD Anderson Cancer Center, Houston, TX, United States

**Keywords:** depression, inflammation, mitochondria, bioenergetics, neurons

## Abstract

Depression is a leading cause of disability and affects more than 4% of the population worldwide. Even though its pathophysiology remains elusive, it is now well accepted that peripheral inflammation might increase the risk of depressive episodes in a subgroup of patients. However, there is still insufficient knowledge about the mechanisms by which inflammation induces alterations in brain function. In neurodegenerative and neuroinflammatory diseases, extensive studies have reported that inflammation negatively impacts mitochondrial health, contributing to excitotoxicity, oxidative stress, energy deficits, and eventually neuronal death. In addition, damaged mitochondria can release a wide range of damage-associated molecular patterns that are potent activators of the inflammatory response, creating a feed-forward cycle between oxidative stress, mitochondrial impairment, inflammation, and neuronal dysfunction. Surprisingly, the possible involvement of this vicious cycle in the pathophysiology of inflammation-associated depression remains understudied. In this mini-review we summarize the research supporting the association between neuroinflammation, mitochondrial dysfunction, and bioenergetic failure in inflammation-associated depression to highlight the relevance of further studies addressing this crosstalk.

## Inflammation-Associated Depression

Research on the communication between the brain and the periphery has shed some light on how the peripheral immune system governs our behaviors and emotions. The existence of intricate neuroimmune interactions is now well recognized by researchers and clinicians and has been widely explored in the context of inflammation-induced sickness behavior and inflammation-induced depression (Yirmiya, 1996; Capuron et al., 2004; Dantzer et al., 2008; Bullmore, 2018). Over the last decades, there has been much evidence indicating that systemic inflammation leads to depression-like behavior in rodents (Yirmiya, 1996; Frenois et al., 2007; Moreau et al., 2008; O’Connor et al., 2009) and to symptoms of depression in otherwise healthy volunteers (Capuron et al., 2004; Brydon et al., 2008; Majer et al., 2008; Eisenberger et al., 2010). This does not mean that all cases of depression are associated with inflammation but it indicates that inflammation-associated depression that is often seen in treatment-resistant depression should be treated as a subgroup of major depression in order to improve the search for more effective therapies (Miller and Raison, 2016; Arteaga-Henriquez et al., 2019).

There have been many studies aiming to test the ability of anti-inflammatory drugs ranging from non-steroidal anti-inflammatory drugs to cytokine antagonists to treat depression as an adjunct to classical antidepressants. However, the results are not always easy to interpret due to insufficient consideration of the efficacy of these drugs on their primary target (inflammation) and the lack of selection of the type of depression to be treated (ideally the subtype of depression associated with inflammation) (Miller and Raison, 2016). In addition, therapeutic progress is still hampered by insufficient knowledge about the mechanisms by which inflammation alters brain function. Most of the work in this field has focused on the monoaminergic theory of depression by showing that inflammation negatively impacts the synthesis, packaging, storage, and release of monoaminergic neurotransmitters (De La Garza and Asnis, 2003; Müller and Schwarz, 2007; Raison et al., 2010; Zhu et al., 2010; Felger et al., 2013; Felger, 2018). Research in the related fields of neurodegenerative and neuroinflammatory diseases has revealed a negative impact of inflammation on mitochondrial function and neuronal health. This negative impact is mainly a consequence of increased reactive oxygen species (ROS) production, oxidative damage to biomolecules, glutamate toxicity, and inflammasome activation (Witte et al., 2010; Park et al., 2015; Bader and Winklhofer, 2019; Joshi et al., 2019). Therefore, inflammation-induced neuronal mitochondrial dysfunction could provide another framework for understanding the pathophysiology of inflammation-associated depression.

Mitochondria arose around two billion years ago from the engulfment of an α-proteobacterium by a precursor of the modern eukaryotic cell and arguably are the reason for the origins of eukaryotes (Lane, 2006). Mitochondria are responsible for adenosine triphosphate (ATP) synthesis by oxidative phosphorylation, intracellular Ca^2+^ homeostasis, generation of free radicals, steroid synthesis, and apoptotic cell death (Picard and McEwen, 2014). The brain, skeletal muscles, and cardiac muscles have high aerobic activity and mitochondrial content and, therefore, are particularly affected by mitochondrial defects (Berg et al., 2002). In the brain, mitochondria are essential for cell growth (Iwata et al., 2020), neurotransmission (Li et al., 2004), maintenance of cell membrane ionic gradients (Devine and Kittler, 2018), synaptic pruning (Ertük et al., 2014), and regulation of inflammation and cytotoxicity (Culmsee et al., 2019).

Considering the extensive role of mitochondria in neuronal tissue, impaired mitochondrial function can have important repercussions on cellular metabolic processes. Therefore, the present mini-review aims to summarize the research around the hypothesis that the symptoms of inflammation-associated depression might stem from functional or quantitative alterations in mitochondria.

## The Crosstalk Between Neuroinflammation and Mitochondrial Dysfunction

Systemic inflammation caused by activation of monocytes by psychological stressors, altered gut permeability, microbial infection, and autoimmune diseases does not remain localized at the periphery but can propagate to the brain by multiple immune-to-brain communication pathways leading to activation of microglia (Dantzer, 2018). As the primary immune effector cells in the central nervous system (CNS), microglia orchestrate neuroinflammation by producing and releasing important immune mediators, including chemokines, proinflammatory cytokines, glutamate, prostaglandins, and ROS (Garden and Möller, 2006; Takeuchi et al., 2006; Hanisch and Kettenmann, 2007). These molecules signal to astrocytes, endothelial cells, and perivascular macrophages to amplify and propagate the inflammatory response resulting in the accumulation of potentially neurotoxic substances in the CNS. In this way and most importantly, microglia-derived signaling molecules can negatively impact mitochondrial function in adjacent cells.

For instance, nitric oxide (NO) produced by inflamed microglia or astrocytes inhibits mitochondrial respiration of surrounding neurons by the reversible inhibition of cytochrome *c* oxidase, which leads to mitochondrial depolarization, ATP depletion, and glutamate release from neurons (Brown and Cooper, 1994; Brorson et al., 1999; Bal-Price and Brown, 2001). NO also affects mitochondrial motility (Zanelli et al., 2006) and dynamics (Eisner et al., 2018), which poses significant detrimental effects for neuronal survival (Schwarz, 2013). Particularly, NO induces mitochondrial fission mediated by dynamin-related protein 1, which is associated with bioenergetic failure and free radical generation that precedes neuronal death (Barsoum et al., 2006).

Upon reacting with superoxide radical anions, NO generates peroxynitrite, which irreversibly inhibits electron transport chain (ETC) complexes I and II and impairs activity of the mitochondrial ATP synthase (Radi et al., 1994; Cassina and Radi, 1996). In particular, it has been shown that the conditioned medium of activated microglia inhibits the ETC complex IV, decreases the mitochondrial membrane potential, and induces a rapid drop in intracellular ATP levels in neurons, an effect dependent on glutamate and *N*-methyl-D-aspartate receptor (NMDAR) activation (Takeuchi et al., 2005). While low concentrations of NO inhibit oxidative energy production, high concentrations of NO inhibit aerobic glycolysis as well (Erecinńska et al., 1995). Taken together, these studies suggest that neurons might suffer from energy depletion during inflamed depression.

Activated glial cells also release proinflammatory cytokines that increase the activity of indoleamine 2,3-dioxygenase, an enzyme involved in the synthesis of kynurenine (Dantzer et al., 2008). One of the kynurenine metabolites is quinolinic acid, which increases glutamate release, blocks its reuptake by astrocytes (Guillemin, 2012), and acts as an NMDAR agonist. Moreover, tumor necrosis factor alpha (TNF-α) stimulates extensive microglial glutamate release in an autocrine manner by upregulating the enzyme glutaminase (Takeuchi et al., 2006). Stimulation of NMDARs triggers Ca^2+^ influx into neurons, which depolarizes the mitochondrial membrane and increases the generation of ROS that can lead to cell death depending on mitochondrial pathways (Hardingham et al., 2002; Qiu et al., 2013; Depp et al., 2018; Mira and Cerpa, 2021).

Overall, chronic ROS exposure can lead to oxidative damage to cellular and mitochondrial proteins, lipids, and DNA. Mitochondrial DNA (mtDNA) is susceptible to the effects of ROS generated by the ETC (Kauppila et al., 2017). Because mtDNA encodes for 13 polypeptides that are subunits of the ETC, accumulation of damage at the level of mtDNA leads to further impairment of oxidative phosphorylation (Nissanka and Moraes, 2018). ROS can inactivate iron-sulfur centers of complexes I, II, and III of the ETC and oxidize thiol groups on the adenine nucleotide translocator, resulting in decreased mitochondrial energy production and formation of the mitochondrial permeability transition pore (Rottenberg and Hoek, 2017).

It is important to note that mitochondrial damage can by itself fuel inflammation, with the potential of forming a highly vicious cycle (West and Shadel, 2017). Damaged mitochondria can release damage associated molecular patterns (DAMPs) into the cytoplasm or the extracellular space, such as mtDNA, ATP, mitochondrial transcription factor A, cytochrome c, and cardiolipin, which activate a wide range of cell surface and intracellular receptors to initiate an immune response (Grazioli and Pugin, 2018). DAMPs can induce a potent inflammatory response mediated by interleukins and type I interferons by activating Toll-like receptors (Lehnardt et al., 2008), receptor for advanced glycation end products (RAGE) (Franklin et al., 2018), inflammasomes (Fleshner et al., 2017), and the cyclic GMP-AMP synthase (cGAS)-stimulator of interferon genes (STING) pathway (Sliter et al., 2018). Further, there is evidence that activated microglia release fragmented mitochondria that propagate inflammation to astrocytes and neurons and can lead to impaired ATP production and reduced mitochondrial inner membrane potential in neuronal cells (Joshi et al., 2019).

Taken together, these observations indicate that inflammation can impair mitochondrial function while dysfunctional mitochondria might drive and/or propagate inflammatory responses, creating a vicious cycle that can compromise neuronal function at the bioenergetic level. Clinical and preclinical studies in biological psychiatry have gathered evidence in favor of mitochondrial dysfunction in major depression, but these results have to be interpreted with caution due to the lack of consideration of the type of depression under study.

## Clinical Evidence for Mitochondrial Dysfunction in Depression

Multiple lines of evidence indicate that mitochondrial processes are altered in patients with major depression. Depressed patients show mtDNA mutations (Gardner et al., 2003; Munakata et al., 2007), higher cell-free mtDNA levels (Lindqvist et al., 2018; Trumpff et al., 2019), altered mtDNA copy number (Chang et al., 2015; Chung et al., 2019), and decreased mitochondrial function (Hroudová et al., 2013; Karabatsiakis et al., 2014) in the periphery. Platelets (Hroudová et al., 2013) and peripheral blood mononuclear cells (Karabatsiakis et al., 2014) of depressed patients showed significantly lower basal and maximal mitochondrial respiration when compared to healthy controls. Reduced ATP production was also detected in muscle biopsies of depressed patients compared to control subjects (Gardner et al., 2003). Besides, a study of 36 patients with mitochondrial disorders reported a lifetime diagnosis of 54% for major depressive disorder, with symptoms of depression preceding diagnosis of the mitochondrial disorder by an average of 7.5 years (Fattal et al., 2007).

Clinically depressed patients also showed reduced glucose utilization in the prefrontal cortex, anterior cingulate gyrus, and caudate nucleus (Videbech, 2000). In most brain regions of these patients, decreased metabolic activity (Mayberg et al., 1994; Newton et al., 2002) and mitochondrial ATP production (Baxter et al., 1989) were observed and might be interpreted as energy deficits. Alterations in ETC complex I and increased oxidative damage were reported in the prefrontal cortex of depressed patients (Ben-Shachar and Karry, 2008). Moreover, 16 mitochondrial genes known to control the production of neuronal ATP, Ca^2+^ handling, and oxidative stress were found to be differentially expressed in the prefrontal cortex of patients with depressive disorders when compared to healthy subjects (Wang and Dwivedi, 2016). Further, mood disorders are often prevalent years before the onset of motor and cognitive symptoms in patients with neurodegenerative diseases, such as Huntington’s, Parkinson’s, and Alzheimer’s disease, in which a major hallmark of the pathophysiology is neuronal mitochondrial dysfunction (Woolley et al., 2011).

Taken together, these studies indicate that while depression is not a classic mitochondrial disease, the link between mitochondrial dysfunction and depression deserves further investigation. More importantly, they highlight the need for comprehensive research addressing mitochondrial function specifically in patients with inflamed depression.

## Preclinical Evidence for Mitochondrial Dysfunction in Inflammation-Induced Depression

While the negative impact of neuroinflammation on mitochondrial function has been extensively explored in animal models of neurodegenerative and neuroinflammatory diseases, it remains understudied in the field of inflammation-associated depression (Table 1). Intraperitoneal injection of a low-dose of lipopolysaccharide (LPS; 0.3–0.83 mg/kg) is widely used to induce depression-like behavior peaking 20–24 h after the injection, which can be assessed by increased immobility time in tests of behavioral despair, decreased sucrose preference, and reduced incentive motivation (Henry et al., 2008; O’Connor et al., 2009; Vichaya et al., 2014). Using this model, a study showed that LPS-treated mice presented increased mitochondrial production of superoxide in the hippocampus, whereas ATP production and mitochondrial membrane potential were decreased (Chen et al., 2017). Stereotaxic administration of mito-TEMPO (a mitochondria-targeted antioxidant with superoxide scavenging properties) or intraperitoneal administration of resveratrol (a natural polyphenol with antioxidant properties), reversed the effects of LPS on mitochondrial function and depression-like behavior (Chen et al., 2017).

**TABLE 1:** Summary of some of the studies showing mitochondrial dysfunction in mouse models of peripheral inflammation-induced depression-like behavior.

**Sex/species**	**Model**	**Brain region**	**Outcome**	**References**
Male ICR mice	LPS, 0.8 mg/kg, intraperitoneal once	Hippocampus	↑ mitochondrial superoxide production and membrane potential ↓mitochondrial ATP	Chen et al., 2017
Male and female Wistar rats	LPS, 0.5 mg/kg, intraperitoneal for 7 days	Prefrontal cortex	↑ COX-1/3 mRNA in males	Brkic et al., 2019
Male and female Wistar rats	LPS, 0.5 mg/kg, intraperitoneal for 7 days	Hippocampus	↓ COX-1/3 mRNA in females	Brkic et al., 2020
Male NMRI mice	DNBS, 6 mg, intra-rectally once	Hippocampus	↑ ROS and nitrite levels ↓ ATP and GSH	Haj-Mirzaian et al., 2017

*Please note that this table is not a summary of all the results described in each study, it only shows those associated with mitochondrial function. ATP, adenosine triphosphate; COX-1/3, cytochrome *c* oxidase 1 and 3; DNBS, dinitrobenzene sulfonic acid; GSH, reduced glutathione; LPS, lipopolysaccharide; ROS, reactive oxygen species.*

In male rats, a 7-day treatment with LPS (0.5 mg/kg) reduced sucrose preference and was accompanied by increased mRNA levels of cytochrome oxidase 1 (COX-1) and 3 (COX-3) in the prefrontal cortex (Brkic et al., 2019). In addition, a study conducted by the same group found that the 7-day treatment with LPS (0.5 mg/kg) decreased mRNA levels of COX-1 and COX-3 in the hippocampus of female rats (Brkic et al., 2020).

Another study used an acute model of colitis to induce depression- and anxiogenic-like behaviors in mice (Haj-Mirzaian et al., 2017). Three days after the intrarectal injection of dinitrobenzene sulfonic acid (DNBS) to induce colitis, adult male mice showed depression-like behavior and decreased levels of reduced glutathione and ATP, increased levels of NO, and increased levels of ROS in the hippocampus.

## Bioenergetic Failure in Dopaminergic Neurons in Inflammation-Associated Depression

The symptomatology of patients with inflamed depression is mostly characterized by motivational changes, anergia, and motor slowing (Jokela et al., 2016), reflecting reduced signaling from the dopaminergic system (Tansey and Goldberg, 2010). Impaired dopaminergic neurotransmission is not considered a core neurochemical alteration of depression; however, there is now much evidence that inflammation preferentially affects midbrain dopaminergic neurons (Hunter et al., 2007; Qin et al., 2007; Felger and Treadway, 2017), for example by reducing dopamine synthesis (Kitagami et al., 2003; Felger et al., 2013) and release (Wu et al., 2007) and increasing dopamine reuptake (Moron et al., 2003).

**FIGURE 1:**
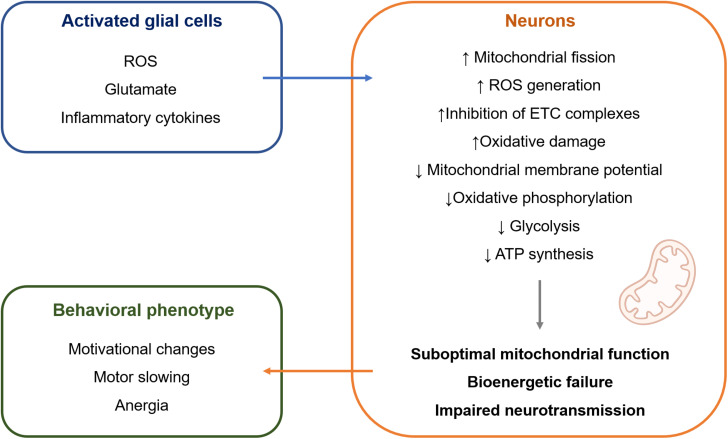
Hypothetical model of intercellular crosstalk during inflammation-associated depression. Activated glial cells release a wide range of molecules (Garden and Möller, 2006; Takeuchi et al., 2006; Hanisch and Kettenmann, 2007) that can impair mitochondrial structure and function in adjacent neurons (Brown and Cooper, 1994; Erecinńska et al., 1995; Brorson et al., 1999; Bal-Price and Brown, 2001; Takeuchi et al., 2005; Zanelli et al., 2006; Eisner et al., 2018; Joshi et al., 2019). It is suggested that this causes bioenergetic failure in neurons, leading to impaired neurotransmission and behavioral manifestation of inflammation-associated depression mainly in the form of motivational deficit, motor slowing, and anergia. ATP, adenosine triphosphate; ETC, electron transport chain; ROS, reactive oxygen species.

The mechanisms that are responsible for this selective susceptibility are not fully understood but might involve particular features of mitochondria in dopaminergic neurons. First, dopaminergic neurons are under constant oxidative stress due to the auto-oxidation of dopamine (DA) that gives rise to dopamine-quinones and superoxide radicals. Second, borrowing from the research on Parkinson’s disease, the selective loss of dopaminergic neurons from the substantia nigra pars compacta (SNc) can be partially explained by their high basal rate of mitochondrial oxidative phosphorylation and high density of axonal mitochondria, that are accompanied by elevated ROS production (Pacelli et al., 2015). Contrasting with mitochondrial dysfunction leading to neuronal death, such as in the context of Parkinson’s disease, it is possible that mitochondrial dysfunction leading to bioenergetic inefficiency might underlie the symptoms of depression (Figure 1). Neurons mainly generate their energy by oxidative phosphorylation (Camandola and Mattson, 2017). Therefore, mitochondrial damage is particularly deleterious for neuronal metabolism. Because dopaminergic neurons are under sustained oxidative stress and high bioenergetic demand, they are more vulnerable to cellular stress, such as the one that happens when mitochondrial dysfunction is induced by chronic inflammation. This would result in suboptimal mitochondrial function and decreased ATP production, which could result in hypoactivity of the dopaminergic system that translates into the symptoms of motivational deficit, anergia, and motor slowing (Dantzer et al., 2021). Indeed, impaired neuronal oxidative phosphorylation was observed during CNS inflammation in the experimental autoimmune encephalomyelitis model, which directly contributed to neuronal vulnerability (Rosenkranz et al., 2021). The same study showed that improving mitochondrial function in neurons by upregulation of *Ppargc1a*, which encodes for peroxisome proliferator-activated receptor gamma coactivator 1-alpha (PGC-1α), one of the master regulators of mitochondrial numbers and function, counteracted the effects of CNS inflammation.

Even though there is evidence for mitochondrial damage in the periphery and CNS of depressed patients and animal models of depression, the important question remains whether mitochondrial dysfunction is a cause or simply an accompanying feature of the behavioral phenotype. It seems unlikely that a single signaling pathway is responsible for the constellation of symptoms experienced by patients with depression. Nonetheless, it appears feasible that the symptoms of inflammation-associated depression are mediated, at least in part, by neuron-intrinsic effects related to mitochondrial bioenergetic dysfunction.

## Sex Differences in Mitochondria Function in Inflammation-Associated Depression

Women have twice the incidence of depression than men [American Psychiatric Association (APA), 2013]. This might also be the case for inflammation-associated depression, since in preclinical and clinical studies, females appear to be more vulnerable to the central effects of peripheral inflammation (Tonelli et al., 2008; Eisenberger et al., 2009; Moieni et al., 2015).

However, research on sex differences in mitochondrial function has yielded mixed results. In rodents, female mice show increased ETC activity, ATP production, and fatty acid utilization, whereas mitochondria from male mice show increased ROS production, mitochondrial biogenesis, protein utilization, and Ca^2+^ uptake (Ventura-Clapier et al., 2017). In response to chronic peripheral administration of LPS, a recent study found that female, but not male mice developed impaired respiration of synaptosomal mitochondria (Shaw et al., 2020). Also, female mice treated with LPS had fewer mitochondria per pre-synaptic terminal as compared to controls and showed a high incidence of mitochondria with broken membranes and cristae, which were not observed in male mice. Knockdown of sirtuin 1, a nicotinamide adenine dinucleotide (NAD^+^)-dependent class III histone deacetylase involved in mitochondrial biogenesis and turnover, energy metabolism, and stress response, in cortical and hippocampal glutamatergic neurons resulted in depression-like behaviors in male but not in female mice (Lei et al., 2020). In humans, mitochondrial complexes I, I + II, and IV, uncoupled respiration, ETC capacity, and ATP levels in PBMCs were significantly higher in females compared to males (Silaidos et al., 2018). Activity of citrate synthase (an enzyme of the mitochondrial Krebs cycle strongly associated with mitochondrial content) was higher in females than in males, suggesting that men have a generally lower mitochondrial function compared to women. The same study also found that the concentration of *N-*acetyl aspartate (a marker of neuronal energy consumption) was significantly higher in the white matter and gray matter of female participants compared to males. However, two studies examining sex differences in cellular respiration of human platelets found no significant differences between males and females (Hroudová et al., 2013; Sjövall et al., 2013). Appropriate inclusion of both sexes in future studies will help fill significant knowledge gaps and perhaps provide another framework to understand the high incidence of depression in women.

## Conclusion

The emerging field of immunopsychiatry has contributed to our understanding on how alterations in the immune system might lead to depression in a subpopulation of patients. Even though not every depressed patient is inflamed, identifying and understanding inflammation-associated depression as a subtype of depression might help with the development of more effective therapeutic interventions. However, better treatment options can only be available after we gain a better understanding of the mechanisms by which inflammation leads to mood and behavioral alterations. Although the literature reviewed here is essentially correlative, the available studies point to an association between mitochondrial dysfunction in inflamed depression that deserves further investigation. Data from neurodegenerative and neuroinflammatory diseases show clearly that neuroinflammation impacts mitochondrial function at different levels. Conversely, DAMPs released by damaged mitochondria are potent inducers of inflammatory responses and might help to propagate neuroinflammation. This results in a potentially vicious cycle that can lead to bioenergetic deficits and impaired neuronal signaling specially in dopaminergic neurons that are under sustained oxidative stress and have high energy demands, ultimately leading to depressive symptoms. It seems intuitive that impaired mitochondrial bioenergetics underlie the symptoms of reduced incentive motivation, anergia, and motor slowing that are present in patients with inflamed depression. However, there are still significant knowledge gaps to be addressed before mitochondrial health can be efficiently targeted by new therapies to relieve symptoms of depression in patients with elevated levels of biomarkers of inflammation. Understanding the pathophysiology of depression is urgent, especially at this time in which the COVID-19 pandemic has affected more than 225 million people worldwide with many of the survivors presenting with long COVID-19. The cases of depression might drastically increase, not only because of the mental constraints imposed by lockdown, social distancing, personal and financial losses, but also because of perturbations of the immune-to-brain communication pathways caused by the infection and/or the neurotropic activity of the virus itself (Mazza et al., 2020; Steardo et al., 2020).

## Author Contributions

AC drafted the manuscript. RD and CB-O edited and reviewed the manuscript. All authors approved the final version of the manuscript.

## Conflict of Interest

The authors declare that the research was conducted in the absence of any commercial or financial relationships that could be construed as a potential conflict of interest.

## Publisher’s Note

All claims expressed in this article are solely those of the authors and do not necessarily represent those of their affiliated organizations, or those of the publisher, the editors and the reviewers. Any product that may be evaluated in this article, or claim that may be made by its manufacturer, is not guaranteed or endorsed by the publisher.
